# Considerations on the regulation of AI systems in the financial sector by the AI Act

**DOI:** 10.3389/frai.2023.1277544

**Published:** 2023-11-10

**Authors:** Gabriele Mazzini, Filippo Bagni

**Affiliations:** ^1^AI Act, European Commission, Brussels, Belgium; ^2^IMT School for Advanced Studies of Lucca, Lucca, Italy

**Keywords:** Artificial Intelligence Act, financial sector, financial institution, bank, creditworthiness, credit scoring

## Abstract

The proposal for the Artificial Intelligence regulation in the EU (AI Act) is a horizontal legal instrument that aims to regulate, according to a tailored risk-based approach, the development and use of AI systems across a plurality of sectors, including the financial sector. In particular, AI systems intended to be used to evaluate the creditworthiness or establish the credit score of natural persons are classified as “high-risk AI systems”. The proposal, tabled by the Commission in April 2021, is currently at the center of intense interinstitutional negotiations between the two branches of the European legislature, the European Parliament and the Council. Without prejudice to the ongoing legislative deliberations, the paper aims to provide an overview of the main elements and choices made by the Commission in respect of the regulation of AI in the financial sector, as well as of the position taken in that regard by the European Parliament and Council.

## 1. Introduction

This paper aims to illustrate the approach of the Commission proposal for the Artificial Intelligence Act (AI Act)^1^ in respect of the regulation of AI systems in the financial sector.^2^ In fact, pursuant to Annex III, point 5, letter (b) of the AI Act, AI systems intended to be used to evaluate the creditworthiness or establish the credit score of natural persons are classified as “high-risk AI systems” and are therefore made subject to a number of important provisions of the AI Act.

The paper is structured as follows: Section 2 provides a short overview of the algorithmic technology that is used by banks in the context of credit scoring; Section 3 provides some background on the key elements and choices of the AI Act, including the key provisions that are relevant for the financial sector; Section 4 contains a short high-level summary of some feedback on the AI Act proposal from the financial sector; Section 5 contains an overview of the position taken in respect of those same provisions by the European Parliament and the Council in their negotiation mandate; Section 6 contains some concluding remarks.

## 2. Overview on the use of AI in the banking/finance sector

Since the non-performing loans crisis of 2008, banking institutions have increasingly developed automated systems to improve their financial services, making significant and growing investments in the application of machine learning algorithmic techniques. In particular, a recent survey initiated by the Commission on the impact of AI tools on European businesses has revealed that financial intermediaries, along with companies in the IT and telecommunications sectors, are the primary users of automated tools for both their external business activities and internal organizational and governance arrangements.^3^

The variety of uses of artificial intelligence in the banking-financial sector can be roughly organized into three main categories.^4^

The first category relates to AI systems that impact the accessibility of financial services for end customers. These systems, like for instance for the purpose of credit scoring or life and health insurance, typically have may have a direct impact on the fundamental rights of individuals, such as the right to housing or health.

The second category is that of AI systems employed with a view to provide personalized financial services to individuals. Examples can include investment advisory services or personalized recommendations for financial products or services. While these systems in principle do not have a direct impact on the enjoyment and access to essential services such as credit or housing, they are also primarily based on customer profiling models that classify individuals based on personal information.

The third category pertains to AI systems that relate essentially to purely economic interests of the customers or the economic operator and do not in principle any direct or indirect impacting on individuals' fundamental rights. Examples belonging to this category can include AI systems for high-frequency trading, for the conduction of stress tests and management of capital requirements or for the orientation of pricing strategies.

Among all the possible applications of AI in the financial sector, in its proposal the Commission chose to focus on creditworthiness assessments and credit scoring,^5^ which were classified as high-risk in Annex III, 5(b) (see also Recital 37).^6^

The significance of credit scoring applications in the banking system is not hard to grasp. The prediction of consumer defaults in financial services is of fundamental importance for banks to correctly select potential borrowers, assess the terms of new loans, and manage associated risks. In recent years, with the increased availability of large datasets and unstructured information, the banking sector has placed growing emphasis on research into machine learning techniques with a view to improve predictive accuracy and limit risks. The added value of these techniques lies not only in improving decision-making in concrete instances, but also in learning from past experiences, enabling the bank to make more sustainable and reliable decisions over time.^7^

The need for technological progress in the field of credit scoring was made evident by the 2008 financial crisis, which exposed the limitations of “traditional” rating systems (slow adaptability to economic changes and inadequate modeling of complex non-linear interactions between economic, financial, and credit variables).^8^

New rating models based on algorithmic machine learning techniques differ from traditional ones in three main aspects: (a) allowing intermediaries to gather and use a larger amount of information; (b) extracting non-linear information from variables; (c) estimating the application of multiple models and use only the most accurate one to perform prediction tasks. This latter characteristic of machine learning models is particularly relevant for credit risk applications, albeit at the cost of reduced transparency (e.g., “decision tree” model).^9^

The predictive benefits associated with the use of these techniques are relevant but may also come with certain downsides in terms of potential opacity, errors, discrimination risk, unfair exclusion from credit, and lack of explainability.^10^

## 3. Key provisions of the proposal for the AI Act, including as regards the financial sector

Following the political mandate to propose a binding legal framework on AI^11^ and building upon the preparatory work and analysis of evidence done since 2018, with the extensive involvement of stakeholders, including academics, businesses, non-governmental organizations, Member States and citizens, in April 2021 the European Commission put forward its proposal for the AI Act.

In the light of the problems related to the development and use of AI systems in the Union to be addressed, of the policy objectives to be achieved and of the assessment of the available policy options, the Commission concluded that a horizontal legislative instrument establishing mandatory requirements and obligations for certain AI applications following a proportionate risk-based approach, whereby AI applications are regulated only where strictly necessary to address the risks and with the minimum necessary regulatory burden placed on operators, was the most appropriate course of action.^12^ In terms of legislative technique and approach, the AI Act has been designed according to the logic of the well-known New Legislative Framework type of legislation,^13^ which has extensively been used for many years for the regulation of products, including software-based products that already incorporate AI, such as medical devices.

The risk-based approach at the center of the AI Act aims to tackle the risks posed by AI systems in a differentiated manner, i.e., the higher the risk, the most stringent the regulatory response should be. Such regulatory response ranges from prohibitions for AI systems and practices that pose an unacceptable risk (Title II) to a comprehensive system of ex-ante compliance and certification for AI systems that pose a high risk (Title III), to information and disclosure obligations for AI systems posing transparency related risks (Title IV) and to the possible establishment of voluntary codes of conduct for AI systems that pose minimal or no risks (Title IX).

As regards in particular the category of high-risk AI systems, to which the largest share of the AI Act is devoted, the new rules focus on a number of important aspects.

First of all, common criteria and a risk assessment methodology are introduced to classify as high-risk the AI use cases with demonstrated concerns for safety and/or fundamental rights. In particular, both AI systems that serve as a safety component of a product already regulated by EU law (Annex II) and stand-alone applications that may be used in the context of a plurality of areas with mainly fundamental rights implications can be considered high-risk. With regard to the banking sector, pursuant to Annex III, point 5, letter (b) of the AI Act, AI systems intended to be used to evaluate the creditworthiness or establish the credit score of natural persons are classified as high-risk AI systems in the context of the access to and enjoyment of essential private service, unless those systems are put into service by small scale providers for own use (see also Recital 37).^14^

The proposal further identifies common mandatory requirements that should be fulfilled for any high-risk AI system to be permitted on the Union market. Those requirements relate to data quality and governance, documentation and traceability, provision of information and transparency, human oversight and robustness, cybersecurity and accuracy. In addition, such requirements are complemented by a set of obligations addressed to the economic and non-economic operators, including the providers who place AI system on the EU market or put it into service, the other actors in the value and distribution chain and the users.

The compliance of high-risk AI systems with the requirements is verified through ex-ante conformity assessments procedures (leading to the affixing of the CE mark) and ex-post supervision and market surveillance. As regards the latter in particular, the AI Act foresees that Member States should designate national competent authorities with the task to control the market and investigate issues of non-compliance, including taking corrective measures and inflicting sanctions, in line with the horizontal system of market surveillance established by Regulation (EU) 2019/1020 on market surveillance (Market Surveillance Regulation).^15^

While, in the light of the particular challenges posed by the emerging technologies, the AI Act is the first specific and comprehensive legal framework establishing rules for the development and design of AI in the EU legal order (as well as globally), other provisions of EU law, including non-AI specific principles and rules such as for instance on the protection of fundamental rights, including protection of personal data, product safety, services or liability, already exist and are applicable to AI systems used in the Union.^16^

The existence of those other provisions of EU law, including sectorial specificities, has been specifically taken into account in the contest of the design of the AI Act with a view to ensure a fully consistent approach.^17^

Following the classification of AI systems intended to be used to evaluate the creditworthiness or establish the credit score of natural persons as high-risk AI systems, specific provisions aimed to ensure consistency with the applicable Union's financial services legislation applicable to regulated banking institutions have been introduced.

In particular, when credit institutions regulated by Directive 2013/36/EU^18^ are providers or users of high-risk AI systems,^19^ in order to minimize the compliance activities the AI Act foresees that certain of its provisions are either deemed to be fulfilled when those institutions comply with relevant provisions of that sectorial legislation or may otherwise be complied with jointly or as part of the compliance with relevant provisions of that same sectorial legislation.

The first scenario relates to the obligation for providers to put in place a quality management system [Art. 17(3)] and the obligation for users to monitor the operation of the high-risk AI systems on the basis of the instructions of use [Art. 29(4)].

The second scenario applies, instead, to the providers' obligations for risk management [Art. 9(9)], for keeping the technical documentation [Art. 18(2)] and the logs [Art. 20(2)], for carrying out the conformity assessment procedure [Art. 19(2) and Art. 43(2)], for post-market monitoring [Art. 61(4)] and for reporting of serious incidents [Art. 62(3)]. The same approach applies to the users' obligation to keep the logs [Art. 29(5)].

In addition, with a view to ensure the coherent application and enforcement of the obligations established in the AI Act and of the relevant rules and requirements of the Union financial services legislation more broadly, Art. 63(4) foresees that the authorities responsible for the supervision and enforcement of the Union financial services legislation should be designated as market surveillance authorities within the meaning of the Market Surveillance Regulation.

## 4. Feedback to the AI Act by sectorial stakeholders and authorities

Following the publication of the AI Act proposal, sectorial stakeholders and authorities shared their perspectives on the Commission's draft and on the ongoing legislative deliberations at the European Parliament and the Council. Generally speaking, they overwhelmingly supported the objective of the AI Act to ensure a high level of protection of health, safety and fundamental rights by fostering the uptake of trustworthy AI in the EU and acknowledged the specific considerations in the proposal made for the financial sector (see Section 3). At the same time, the feedback provided emphasized, among other things, the importance of putting in place a balanced and coherent approach, considering the existing legislative sectorial frameworks and of ensuring clarity on the role and responsibilities of relevant supervisory authorities.^20^

When it comes to institutional actors, the authorities that provided explicit feedback on the Commission proposal were the European Central Bank (ECB) and the European Insurance and Occupational Pension Authority (EIOPA).

In its opinion of 29 December 2021,^21^ the ECB referred to its institutional role and prerogatives in the context of prudential supervision of certain credit institutions pursuant to Regulation (EU) 1024/2013,^22^ which would prevent it from exercising the role of marker surveillance authorities within the meaning of the Market Surveillance Regulation and highlighted the need to clearly differentiate between ex-ante conformity assessment procedures and ex-post market surveillance activities in respect of the same credit institutions.^23^

EIOPA emphasized that national and European sectorial authorities should remain responsible for supervising the development and use of AI system in the insurance sector and should also adequately be involved as permanent observers in the AI Board newly established by the AI Act. While speaking against the inclusion of the insurance sector among the high-risk use cases in Annex III, EIOPA nonetheless stressed the importance of overall regulatory consistency considering the existing risk management and governance systems required by sectorial legislation and advocated for the inclusion of cross references similar to those made for the banking sector, for instance as regards quality management system.^24^

While it did not issue a dedicated position statement on the AI Act, in its follow-up report on machine learning for IRB models the European Banking Authority (EBA) included some remarks as regards the possible impact of the AI Act on the use of machine learning techniques in IRB models. Among others, the EBA noted that the use case in Annex III, point 5, letter (b) should be limited only to systems used for creditworthiness assessment and credit scoring of natural person at the point of loan origination to grant the credit or related financial services, and it therefore does not apply directly to other areas of the credit process such as IRB models used for capital requirements calculation. Nonetheless, EBA also observed that the AI Act may produce indirect effects on the IRB models via the prudential use-test requirements. Indeed, it is well-known that internal ratings and default and loss estimates used by financial institutions in the calculation of own funds requirements and associated systems and processes play an essential role in the risk management and decision-making process, and in the credit approval of the institutions.^25^

In the light of that, for the EBA it would therefore be important to avoid inconsistencies and uncertainty as regards the regulatory framework applying to the financial institution's IRB models.^26^

## 5. The position by the Council and the European Parliament

Also in the light of the feedback provided by sectorial stakeholders and institutions, in its General Approach the Council reworked relevant provisions of the AI Act proposal. As regards the role of the ECB, the Council clarified that this institution should not fulfill the tasks and responsibilities of the market surveillance authority within the meaning of the AI Act and the Market Surveillance Regulation, but it established that the ECB should receive any information, identified by national authorities in the course of market surveillance activities, which may be of relevance for the ECB's prudential supervisory tasks.^27^ The Council made clear that, ex-ante conformity assessment being a responsibility of the provider, only the ex-post market surveillance activities of the authorities can be integrated into the existing supervisory mechanisms and procedures under the relevant Union financial services legislation,^28^ such as for instance the Supervisory Review and Evaluation Process (SREP) that is foreseen for the banking sector.^29^ It also specified in this context that the principle whereby the market surveillance authorities for the purposes of AI Act should be the relevant national authorities responsible for the supervision of the financial institutions under applicable Union financial services legislation applies in so far as the placement on the market, putting into service or the use of the AI system is in direct connection with the provision of those financial services [Art. 63(4)]. Building upon the Commission's proposal [cf. Artt. 57(1) and (4)], the Council stressed the importance of ensuring an adequate degree of coordination and collaboration between the AI governance mechanisms and actors established by the AI Act (AI Board) and sectorial authorities {cf. Art. 56(2), [2aa(iii)], Art. 58(f) of the General Approach}. Furthermore, also following the political choice to introduce new high-risk use cases for the insurance sector [cf. Annex III, point 5(d)],^30^ the Council opted to delete in the enacting terms all references to “credit institutions” and specific banking legislation (notably Directive 2013/36/EU) and its relevant requirements regarding internal risk management and governance arrangements and processes and instead make a broad reference to “Union financial services legislation” [see notably Artt. 17(3), 18(2), 20(2), 29(4), 29(5), 61(4), 62(3) and explanation in recital 80 of the General Approach],^31^ along the lines of the approach taken by the Commission in Art. 63(4) as regards the designation of sectorial authorities as market surveillance authorities.

With regard to the opinion of European Parliament, it maintained the Commission's proposal on certain elements. For instance, the Parliament largely kept the references to “credit institutions” and the specific banking legislation Directive 2013/36/EU [see notably Artt. 11(3), 17(3), 20(2), 29(4), 29(5), 43(2) and 61(4)], even if the scope of the AI Act was extended to the insurance sector [see Annex III, 5(b a)].^32^ On other aspects, to the contrary, the Parliament aligned with the Council position: beyond the extension of Annex III to certain use cases in the insurance sector, in Art. 9(9) and in Art. 62(3) the Parliament broadened the reference to providers that are already subject under EU law to, respectively, internal risk management procedures as well as incident reporting obligations, as proposed also by the Council.

Finally, as requested by certain stakeholders arguing that the principle of “same activity, same risks, same rules” must be taken into account, the Parliament deleted, as regards the creditworthiness evaluation and credit score use case in Annex III, 5(b), the exception for providers that are micro and small-sized enterprises as defined in the Annex of Commission Recommendation 2003/361/EC for their own use. It also clearly excluded from that use case AI systems for the purpose of detecting financial fraud.

## 6. Closing remarks

Following the adoption of their respective position on the AI Act, the European Parliament and the Council have just entered into the phase of “trilogies”, during which the co-legislators are expected to find a common ground and come to a mutually agreed upon final text of the AI Act.

Pursuant to the ordinary legislative procedure foreseen in Art. 294 TFEU, a proposal for a legal act put forward by the European Commission shall be adopted jointly by the European Parliament and the Council. The two co-legislators have equal rights and obligations and they have to approve an identical text, which requires time and negotiations. With a view to ensure the effectiveness of the legislative process, “trilogies” have emerged in the practice as one of the most common tools used in that respect. They consist in informal tripartite meetings between representatives of the European Parliament, the Council and the Commission. The Commission does not have a decision-making role and acts solely to provide technical support to the other two institutions in order to facilitate reaching a compromise.^33^

It is impossible at this stage to predict the text of the AI Act on which the co-legislators will ultimately agree, including in respect of the provisions that are of relevance for stakeholders and institutions in the financial sector.

However, one can observe that, although with some variations, Parliament and Council appear to converge on two important points.

On the one hand, they confirm the approach of the Commission to specifically take into account the existence of sectorial legislation applicable to providers and users in the finance sector in broad terms, i.e., beyond the banking sector strictly speaking.^34^ On the other hand, they both extend the list of high-risk use cases to certain AI systems intended to be used in the insurance sector (health and life insurance), unequivocally expanding the scope of concerned financial institutions beyond “credit institutions”.

If these choices are confirmed, and with a view to ensure an even and consistent safeguard of the interests of persons and consumers and an equally even and consistent regulatory treatment of market operators putting into service or using high-risk AI systems in similar conditions, further guidance and specifications could possibly be useful to clarify the scope of the Union financial services legislation referenced in the AI Act and the relevant financial institutions subject to it.

The European legal order does not seem to provide a single and uniform definition of “*financial sector*” or of “*financial institutions*”, but rather seem to contain a plurality of definitions that are spread across multiple legal frameworks.^35^

In addition to clarifying the financial institutions that could be relevant as providers or users in the context of the AI Act beyond the non-credit and non-insurance financial institutions,^36^ it could also be useful to provide more specific guidance on the interplay between sectorial requirements regarding internal governance and risk management rules and the obligations established in the AI Act.

## Author contributions

GM: Writing – original draft, Writing – review & editing. FB: Writing – original draft, Writing – review & editing.

## Footnotes

^1^Proposal for a regulation of the European Parliament and of the Council laying down harmonized rules on artificial intelligence (Artificial Intelligence Act) and amending certain union legislative acts (COM/2021/206 final) (2021). Available online at: https://eur-lex.europa.eu/legal-content/EN/TXT/; The proposal for the AI Act put forward by the Commission is currently being debated by the EU co-legislators: the European Parliament and the Council. The content of the AI Act as finally adopted by the co-legislators may therefore differ from the text that is discussed herein. Unless explicitly stated otherwise, all references to the AI Act in this paper shall be understood as references solely to the proposal by the Commission; The European Parliament adopted his negotiating position on the AI Act on 14.6.2023 (2023). Available online at: https://www.europarl.europa.eu/doceo/document/; The Council adopted his negotiating position on the AI Act on 6.12.2022 (General Approach) (2023). Available online at: https://data.consilium.europa.eu/doc/document/.

^2^Among the emerging pieces of literature on the AI Act, including in relation to the financial sector, see, for instance: Edwards (2012), Floridi (2021), De Gregorio and Dunn (2022), Giudici and Raffinetti (2023), Mazzini and Scalzo (2023), and Sciarrone Alibrandi et al. (2023).

^3^Specifically, the main applications recorded in the financial sector from the survey pertain to fraud management, claims management, customer profiling and segmentation, as well as product and policy design. Cf. European Commission (2021). Study on the Relevance and Impact of Artificial Intelligence for Company Law and Corporate Governance. Available online at: https://op.europa.eu/en/publication-detail/-/publication.

^4^For a more detailed analysis of a taxonomy of AI systems in the financial sector see Sciarrone Alibrandi et al. (2023).

^5^Credit scoring is an automated procedure adopted by banks to assess customers' loan applications. Such procedure mainly involves the application of statistical methods or models to assess credit risk, the results of which are expressed in the form of summary ratings (numerical indicators or scores) associated with the person concerned, aimed at providing a representation, in predictive or probabilistic terms, of the customer risk profile and payment reliability. For a more detailed description of the influence of big data on the assessment of a customer's creditworthiness, see Ferretti (2018). For a more detailed description of the use of algorithms in the European credit market see also Bagni (2021).

^6^Recital 37 explains that those systems “should be classified as high-risk AI systems since they determine those persons' access to financial resources or essential services such as housing, electricity, and telecommunication services. AI systems used for this purpose may lead to discrimination of persons or groups and perpetuate historical patterns of discrimination, for example, based on racial or ethnic origins, disabilities, age, sexual orientation, or create new forms of discriminatory impacts”.

^7^For an up-to-date analysis of the use of machine learning techniques in the context of internal ratings-based (IRB) models see most recently European Banking Authority (2023). Machine learning for IRB models. A follow-up report from the consultation on the discussion paper on machine learning for IRB models. Available online at: https://www.eba.europa.eu/sites/default/documents/files/document_library/Publications/Reports/2023/. See also Domingos (2016) and Kaplan (2016).

^8^Cf. Moscatelli et al. (2019).

^9^A classic example of an algorithm used for credit scoring is the decision tree, in which there is a set of rules that recursively partition the entire customer dataset into homogeneous subsets based on their characteristics and the outcome variable (default/non-default). Predictions are then obtained in the form of probabilities of a given outcome within each subset. Cf. Gambacorta et al. (2019). See also Alloway (2015).

^10^For an overview of some of the challenges related to using machine learning techniques for the development of IRB models and credit risk estimation, including explainability, see: European Banking Authority (2023). Machine learning for IRB models. A follow-up report from the consultation on the discussion paper on machine learning for IRB models. Available online at: https://www.eba.europa.eu/sites/default/documents/files/document_library/Publications/Reports/2023/; Gramegna and Giudici (2021). On the role of explainability in the AI Act see Panigutti et al. (2023).

^11^President Von der Leyen announced a legislation for a coordinated European approach on the human and ethical implications of Artificial Intelligence, in her political guidelines for the 2019-2024 Commission. Available online at: https://commission.europa.eu/system/files/2020-04/political-guidelines-next-commission_en_0.pdf.

^12^For further details, refer to the Commission Staff Working Document (2021). Impact Assessment, accompanying the proposal for the AI Act. Available online at: https://eur-lex.europa.eu/legal-content/EN/TXT/?uri=celex%3A52021SC0084.

^13^For a more detailed description of the relationship between the New Legislative Framework approach and the AI Act, see Mazzini and Scalzo (2023).

^14^Recital 37 explains how certain private services and public services are essential for people to fully participate in society or to improve one's standard of living: “in particular, AI systems used to evaluate the credit score or creditworthiness of natural persons should be classified as high-risk AI systems, since they determine those persons' access to financial resources or essential services such as housing, electricity, and telecommunication services. AI systems used for this purpose may lead to discrimination of persons or groups and perpetuate historical patterns of discrimination, for example based on racial or ethnic origins, disabilities, age, sexual orientation, or create new forms of discriminatory impacts”.

^15^Regulation (EU) No. 2019/1020 of the European Parliament and of the Council of 20 June 2019 on market surveillance and compliance of products and amending Directive No.2004/42/EC and Regulations (EC) No 765/2008 and (EU) No 305/2011 (OJ L 169, 25.6.2019, p. 1).

^16^See Commission Staff Working Document (2021). Impact Assessment, accompanying the proposal for the AI Act. Available online at: https://eur-lex.europa.eu/legal-content/EN/TXT/?uri=celex%3A52021SC0084; For an analysis of the several fields of EU law that are relevant and potentially applicable to AI, see Mazzini (2019). For an overview of the European financial framework relevant for the use of AI systems in the financial sector, see also Sciarrone Alibrandi et al. (2023).

^17^Explanatory memorandum of the proposal for the AI Act. (2022). Available online at: https://eur-lex.europa.eu/legal-content.

^18^Directive No. 2013/36/EU of the European Parliament and of the Council of 26 June 2013 on access to the activity of credit institutions and the prudential supervision of credit institutions and investment firms, amending Directive 2002/87/EC and repealing Directives 2006/48/EC and 2006/49/EC (OJ L 176, 27.6.2013, p. 338).

^19^Art. 63(4) and recital 80 of the AI Act deal not only with the credit institutions regulated by Directive No. 2013/36/EU, but more generally with “regulated financial institutions”.

^20^Stakeholders' groups providing detailed feedback included, for instance, the European Banking Federation (paper dated 27 September 2021) and the European Financial Services Round Table (paper dated June 2022).

^21^Opinion of the European Central Bank of 29 December 2021 on a proposal for a regulation laying down harmonized rules on artificial intelligence (CON/2021/40) (OJ C 115, 11.3.2022, p. 5).

^22^Council Regulation (EU) No 1024/2013 of 15 October 2013 conferring specific tasks on the European Central Bank concerning policies relating to the prudential supervision of credit institutions (OJ L 287, 29.10.2013, p. 63).

^23^For a more detailed analysis of the ECB opinion see also Bagni (2022).

^24^Cf. Letter to the co-legislators on the Artificial Intelligence Act. (2023). Available online at: https://www.eiopa.europa.eu/system/files/2022-07/letter_to_co-legislators_on_the_ai_act.pdf.

^25^See the relevant regulation in Art. 144(1)(b) of the Regulation No. 575/2013 on prudential requirements for credit institutions and investment firms (OJ L 176, 27.6.2013, p. 1).

^26^See page 15 of European Banking Authority (2023). Machine learning for IRB models. A follow-up report from the consultation on the discussion paper on machine learning for IRB models. Available online at: https://www.eba.europa.eu/sites/default/documents/files/document_library/Publications/Reports/2023/.

^27^See art. 63(4) and recital 80 of the General Approach text of the Council.

^28^Deletion of Art. 19(2) and Art. 43(2), second sentence and amended recital 80 of the General Approach of the Council.

^29^The Supervisory Review and Evaluation Process is the procedure whereby the relevant supervisory authorities (ECB for significant banks and National Central Banks for less significant banks) carry out a risk assessment and measurement exercise at the individual bank level, summarizing the results of the analysis for a given year and indicating to the bank the action to be taken.

^30^Annex III, point 5(d): “AI systems intended to be used for risk assessment and pricing in relation to natural persons in the case of life and health insurance”.

^31^While emphasizing the need to ensure that all financial institutions subject to similar requirements regarding internal governance, arrangements or processes according to EU law should be treated equally and consistently as regards their obligations under the AI Act, the recital 80 of the General Approach introduced a specific reference to insurance legislation: “The same regime should apply to insurance and re-insurance undertakings and insurance holding companies under Directive 2009/138/EU (Solvency II) and the insurance intermediaries under Directive 2016/97/EU and other types of financial institutions subject to requirements regarding internal governance, arrangements or processes established pursuant to the relevant Union financial services legislation to ensure consistency and equal treatment in the financial sector”. As regards Art. 9(9), the reference to credit institutions was deleted and replaced by a reference to providers of high-risk AI systems that are already subject under sectorial law to internal risk management procedures.

^32^Annex III, 5(b a): “AI systems intended to be used for making decisions or materially influencing decisions on the eligibility of natural persons for health and life insurance”.

^33^Although not foreseen by the EU treaties, trilogues are one of the most common tools used in that respect. They consist of informal tripartite meetings on legislative proposals between representatives of the European Parliament, the Council and the Commission. The Commission is not a co-legislator and does therefore not have a decision-making role. In the context of those meetings, it solely acts to provide technical support to the other two institutions on a need basis in order to facilitate reaching a compromise. For further details see European Parliamentary Research Service (2021). Understanding trilogue. Informal tripartite meetings to reach provisional agreement on legislative files. Available online at: https://www.europarl.europa.eu/RegData/etudes.

^34^Although the Parliament kept the references to Directive No. 2013/36/EU as regards the providers' and users' obligations, it also maintained the broader reference to Union financial services legislation in Art. 63(4). The Council clearly extended the relevance of internal governance and risk management rules and requirements established in sectorial legislation to several institutions in the insurance sector and to other types of financial institutions (recital 80).

^35^As mere examples, the following definitions can be mentioned: the definition of “financial sector” in Art. 2 of Directive No. 2002/87/EC on the supplementary supervision of credit institutions, insurance undertakings and investment firms in a financial conglomerate (OJ L 35, 11.2.2003, p. 1); the definition of “institution”, “financial institution” and “financial sector entity” in Art. 4 of Regulation No. 575/2013; the definition of “insurance undertaking”, “reinsurance undertaking” and “financial undertaking” in Art. 13 of Directive No. 2009/138 on the taking-up and pursuit of the business of Insurance and Reinsurance (Solvency II) (OJ L 335, 17.12.2009, p. 1); the definition of “financial institution” in Art. 4(1) of Regulation No. 1093/2010 establishing a European Supervisory Authority (European Banking Authority), (OJ L 331, 15.12.2010, p. 12) and Art. 4(1) of Regulation No. 1094/2010 establishing a European Supervisory Authority (European Insurance and Occupational Pensions Authority) (OJ L 331, 15.12.2010, p. 48).

^36^Based on a very preliminary analysis the Union financial services legislation, the concept of ‘financial sector' referred to in the AI Act could include the banking sector (“credit institution” within the meaning of Article 3(1) of Directive 2013/36/EU, which itself refers to the definition in Article 4(1) of Regulation (EU) No 575/2013), the insurance sector (“insurance undertaking”, “reinsurance undertaking”, “insurance holding company” within the meaning of Article 13(1-2-4-5) or Article 212(1)(f) of Directive No. 2009/138/EC and “insurance intermediaries” as defined in Directive No. 2016/97 on insurance distribution (OJ L 26, 2.2.2016, p. 19)), the investment services sector (“investment firm” within the meaning of Article 3(2) of Directive 2013/36/EU, which itself refers to the definition in Article 4(2) of Regulation (EU) No 575/2013) and the mixed financial sector (“financial conglomerates” and “mixed financial holding company” within the meaning of Article 2(14-15) of Directive No. 2002/87/EC).
